# Anticonvulsant effect of glycitin in pentylenetetrazol induced male Wistar rat model by targeting oxidative stress and Nrf2/HO-1 signaling

**DOI:** 10.3389/fphar.2024.1392325

**Published:** 2024-08-23

**Authors:** Saghi Hakimi Naeini, Hassan Rajabi-Maham, Vahid Azizi, Abdolkarim Hosseini

**Affiliations:** Department of Animal Sciences and Marine Biology, Faculty of Life Sciences and Biotechnology, Shahid Beheshti University, Tehran, Iran

**Keywords:** anticonvulsants, epilepsy, glycitin, pentylenetetrazol, soybeans

## Abstract

Epilepsy, characterized by recurrent seizures, poses a significant health challenge globally. Despite the availability of anti-seizure medications, their adverse effects and inadequate efficacy in controlling seizures propel the exploration of alternative therapeutic measures. In hypothesis, glycitin is a phytoestrogenic compound found in soybeans and due to its estrogenic properties may have anti-epileptic and neuroprotective effects. This study investigates the potential anti-epileptic properties of glycitin in the context of pentylenetetrazol (PTZ) induced seizures in male Wistar rats. The rats were pretreated with varying doses of glycitin (5, 10, and 20 mg/kg) before PTZ (35 mg/kg) administration, and assessments included behavioral observations and histological evaluation via hematoxylin and eosin (H&E) staining. Additionally, oxidative stress markers, such as malondialdehyde (MDA), glutathione peroxidase (GPx), and superoxide dismutase (SOD) levels, were quantified to examine glycitin’s impact on oxidative stress. Molecular analysis was conducted to assess the activation of the Nuclear factor erythroid 2-related factor (Nrf2)/Heme oxygenase 1 (HO-1) signaling pathway. Results indicated that glycitin pretreatment effectively mitigated PTZ-induced convulsive behaviors, supported by histological findings from H&E staining. Furthermore, glycitin administration led to significant alterations in MDA, GPx, and SOD levels, suggestive of its ability to modulate oxidative stress. Notably, glycitin treatment induced activation of the Nrf2/HO-1 signaling pathway. These findings underscore the potential of glycitin as an anticonvulsant agent, elucidating its mechanism of action through histological protection, modulation of oxidative stress markers, and activation of the Nrf2/HO-1 signaling pathway.

## Graphical Abstract

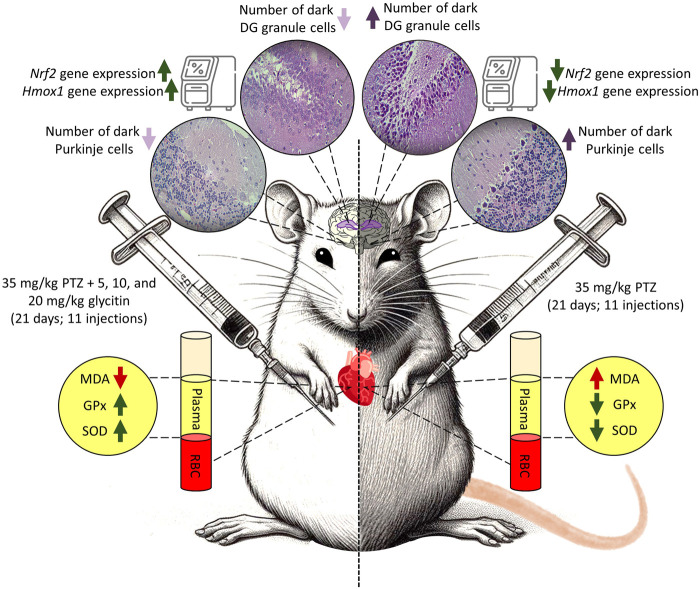

## 1 Introduction

Epilepsy is a complex neurological condition that affects about 0.5%–2% of people worldwide, with a higher prevalence in developing nations (Mohamed and Eltony, 2020; Rajabzadeh et al., 2012; Van Den Broek et al., 2004). It is recognized by recurring unprovoked seizures due to abnormal neuronal electrical activity of brain, caused by a lack of balance between excitation and inhibition (Mohamed and Eltony, 2020). This can affect the nervous system in a limited region and spread to involve cortical-subcortical pathway (Hou et al., 2022; Milton et al., 2007). The exact mechanisms of epileptogenesis are not clearly understood, however, they may involve neuroinflammation, oxidative stress, and dysfunction of the neurotransmitter systems (Wu et al., 2020).

People with epilepsy experience progressive loss of neurological functions, psychosocial disability, and systemic disorders (Tolchin et al., 2020; Yuen et al., 2018). Despite the availability of multiple anti-seizure medications (ASMs) (French and Perucca, 2020), a significant portion of epilepsy patients continues to experience inadequate seizure control and adverse side effects such as depression, psychotic episode, irritability, and aggression (Hixson, 2014; Wang et al., 2021a). Therefore, an urgent need for more exploration of alternative treatment strategies that can better manage epilepsy (Wang et al., 2021a).

Many studies consistently demonstrate the important function of oxidative stress in the progression and development of epilepsy in various kindling animal models (Etemad et al., 2019; Khodayar et al., 2019). The kindling animal model is a method used in research to induce epileptic seizures by repeatedly stimulating a specific area of the brain (Wang et al., 2022). Experimental evidence indicates that oxidative stress can reduce antioxidants such as superoxide dismutase (SOD), glutathione peroxidase (GPx), and increase the level of malondialdehyde (MDA), resulting in disruption of lipid membranes, cellular proteins and DNA (Aguiar et al., 2012; Liu et al., 2019). Nuclear factor erythroid 2-related factor (Nrf2) transcription factor/Heme oxygenase 1 (HO-1, encoded by *Hmox1* gene) is an essential antioxidant signaling pathway that protects against inflammation, apoptosis, and oxidative stress (Han et al., 2019; Upadhayay and Mehan, 2021). Recent research has uncovered the significant neuroprotective effects of the Nrf2/HO-1 pathway in epilepsy, mitigating the detrimental impact of oxidative stress (Liu et al., 2018b). Consequently, the Nrf2/HO-1 pathway has become a focus for potential therapeutic strategies to enhance neuroprotection (Calkins et al., 2009; Khalifeh et al., 2017; Singh and Shekh-Ahmad, 2024; Wang et al., 2021b).

In the last few years, natural plant-derived compounds have garnered considerable attention caused by their potential medicinal properties, including the ability to modulate seizures (Guo et al., 2021; Kazmi et al., 2020; Khodamoradi et al., 2018; Sharma et al., 2020; Zhu et al., 2014). Among these bioactive compounds, glycitin, also known as 4′-hydroxy-6-methoxyisoflavone-7-D-glucoside, is a natural phytoestrogenic isoflavone derived from soybeans and soy-based products (Kim et al., 2015; Patel, 2023). It has been extensively studied for its various biological functions, including antioxidant properties (Seo et al., 2014), anti-obesity and anti-diabetic effects (Zang et al., 2015), prevention of cardiovascular diseases and some types of cancer (Hsu et al., 2010; Robb and Stuart, 2014), prevention of bone diseases (Li et al., 2005; Zhang et al., 2016), alleviation of acute lung injury (Chen et al., 2019), and promotion of wound healing (Seo et al., 2017). Furthermore, glycitin, as a phytoestrogen, may have the potential to influence epilepsy due to its ability to mimic estrogen and act as a neuro-modulator (Afsordeh et al., 2016; Villaseca, 2012). The estrogenic properties of glycitin suggest a possible role in modulating neuronal activity and potentially impacting epileptic seizures (Patel, 2023; Zuo et al., 2020).

There are several experimental models available for studying the mechanisms of acute and chronic epilepsy (Rubio et al., 2010). These models involve the administration of convulsive drugs or electrical stimulation to induce epileptic seizures. By utilizing various chemicals like pentylenetetrazol (PTZ), penicillin, kainic acid, and others, researchers can effectively provoke both partial and generalized seizures (Bambal et al., 2011; Rubio et al., 2010). One routinely used experimental model to investigate seizures is the PTZ induced seizures (Löscher et al., 1991). PTZ, a tetrazole compound, is known to induce generalized tonic-clonic seizure. The mechanism of PTZ involves the disruption of gamma amino butyric acid (GABA), an important inhibitory neurotransmitter (Ciltas et al., 2023). Studies have shown that PTZ induced models exhibit the formation of free radicals during and after seizures (Rauca et al., 1999). In cases of chronic PTZ exposure, the production of free radicals significantly increases and causes oxidative stress damage (Khan et al., 2024). The PTZ model is valuable for accurately recording the onset of seizures, identifying the key events underlying epilepsy, post-seizure dysfunctions and serves as a screening tool for potential treatments (Samokhina and Samokhin, 2018). Furthermore, it allows researchers to study cognitive, physical, and emotional deficits observed in human epilepsy (Lin et al., 2019; Mortazavi et al., 2005; Nasir et al., 2012).

The antiepileptic effects of glycitin have not yet been researched. Further investigation is needed to determine how different doses of this compound may impact seizure control. Therefore, in this study we aimed to bridge this gap of knowledge by evaluating the effects of different doses of glycitin on seizures induced by PTZ in male Wistar rats, focusing on the behavioral alterations, histological analysis and regulation of oxidative stress.

## 2 Materials and methods

### 2.1 Drugs and chemicals

To carry out this study, the drugs purchased and used were glycitin and PTZ (Sigma company, United States), phenobarbital ((PB) manufactured by Chemidarou Pharmaceutical company, Iran), ketamine and xylazine (Alfasan Company, Netherlands). Dimethyl sulfoxide (DMSO) and Dulbecco’s modified Eagle’s medium (DMEM) were bought from Merck, Germany. The thiobarbituric acid (TBA), SOD, and GPx assay kits were bought from Kiazist, Iran. Total RNA Extraction, cDNA Reverse Transcription kits, and 2X SYBR Green Plus Real Time PCR Master Mix were bought from Parstous company, Iran. All the other chemicals and reagents were bought from reputed manufacturers of analytical grade.

### 2.2 Animals and ethical statement

The present study was conducted on male Wistar rats at Shahid Beheshti University in Tehran, Iran. The rats were obtained from Shahid Beheshti Medical University, weighed between 220 ± 20 g, and were 8 weeks old. To ensure their adaptation to their new environment, the rats were held in standard polycarbonate cages (38 cm × 23 cm × 10 cm) with 4 animals per cage under controlled conditions for 1 week (Mehla et al., 2010). These conditions included a 12-h light-dark cycle with lights turning on at 7:00 a.m. and off at 7:00 p.m., temperature at 22°C ± 2°C, and humidity of 55% ± 5%. Rats had regular access to water and diet (Pars animal food, Iran) during the whole experiment. All necessary precautions were taken to ensure the animals’ wellbeing and minimize any potential discomfort or pain. Based on simple randomization (Fowler and Fleming, 2023) the rats were divided into distinct experimental groups, there were 6 rats in each group, and throughout the experiment each animal was used only once. All procedures adhered to the ARRIVE guidelines 2.0 and the guidelines outlined in the European Communities Council Directive of 2010/63/EU, which were approved by the Ethics Committee at the University of Shahid Beheshti (ethical code: IR. SBU.REC.1402.152).

### 2.3 Experimental design

**FIGURE 1:**
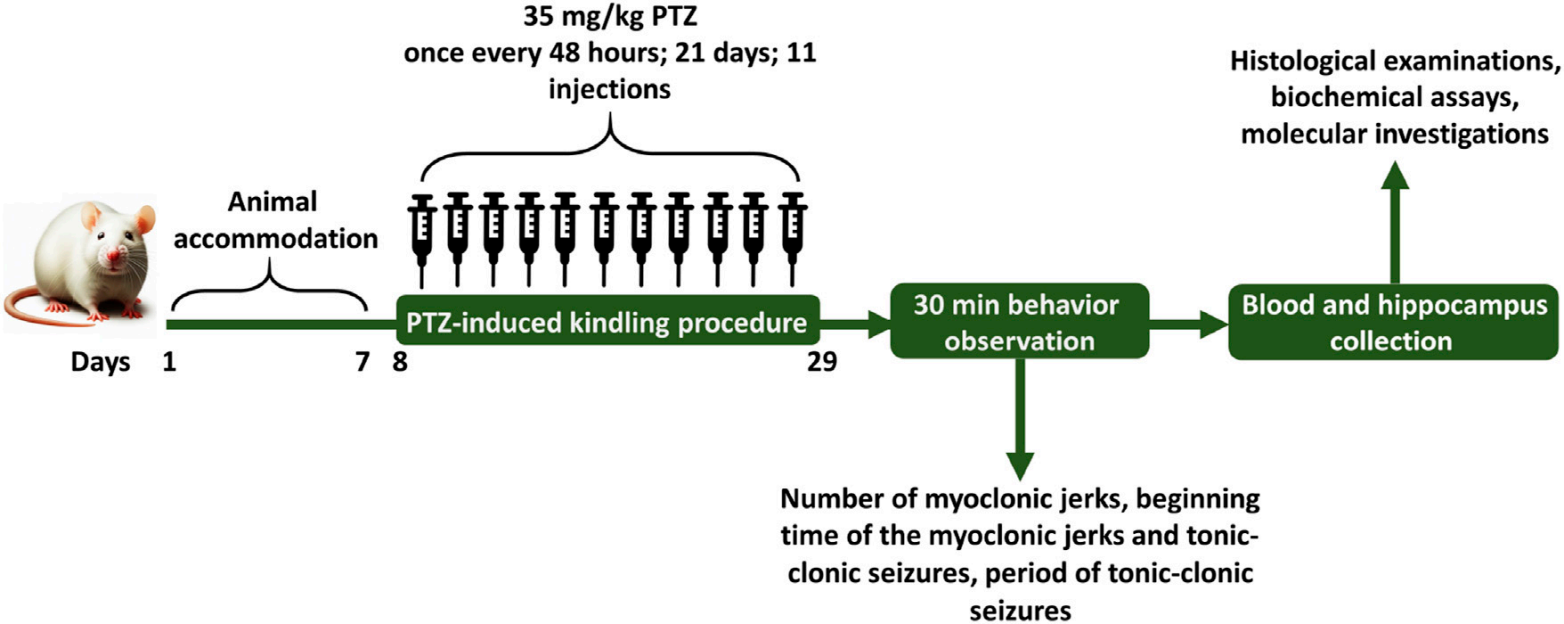
The schedule for the experiment and the intervals for estimating different parameters.

Animals were divided into 6 groups by simple randomization method, each group included 6 rats. These groups as follows: 1) vehicle control (VC) group receiving equal amount of 1% DMSO in physiological saline and DMEM (Zhao et al., 2023) initially, followed by physiological saline (0.3 mL/rat) after 30 min, 2) control negative (CN) or PTZ treated group receiving equal amount of 1% DMSO in physiological saline and DMEM initially, followed by the subthreshold dose of PTZ (35 mg/kg) after 30 min, 3) control positive (CP) group receiving PB (30 mg/kg) 30 min before the PTZ injection, (4, 5 and 6) groups receiving different doses of glycitin (5, 10, and 20 mg/kg) respectively, followed by PTZ after 30 min. Glycitin, purchased in powder form, was prepared to be dissolved in 1% DMSO (Argenta et al., 2015) and diluted with DMEM (Chen et al., 2019; Zhao et al., 2023) and was given at the mentioned appropriate doses (Chen et al., 2019). PTZ was used in a sterile saline at 0.9% W/V solution and PB was dissolved in 1% DMSO (Eyal et al., 2004) and diluted with DMEM. Experiments were carried out from 9:00 a.m. to 15:00 p.m. and all injections were given intraperitoneally (i.p) once every 48 h for a total of 21 days and the injection volume was 0.3 mL/rat (Kazmi et al., 2020). The schedule for the experiment and the intervals for estimating different parameters are illustrated in Figure 1.

### 2.4 Epileptic seizures monitoring

Behavioral characterization was measured based on the modified Racine’s scale (Table 1) (Terzic et al., 2021) and PTZ injections were given every other day until the animals experienced two consecutive score 4 and 5 seizures, indicating the creation of a kindled model (Azizi et al., 2022; Vasilev et al., 2018). Following the final injection of rats with PTZ, animals were transferred individually to a clear plastic box (40 cm × 40 cm × 80 cm) for recording and evaluation of epileptic behavior. Shortly after completing the PTZ challenge, epilepsy behavior was detected for 30 min and recorded on the computer via a connected camera. The behaviors that were measured in this study include: the number of myoclonic jerks, the latency to the myoclonic jerks and tonic-clonic seizures, and also the duration of tonic-clonic seizures. Myoclonus refers to rapid, sudden, shock-like involuntary jerking movements affecting small muscles or the entire body (Tai and Truong, 2015) and tonic-clonic seizures refers to muscle stiffening (tonic phase), followed by rhythmic jerking movements (clonic phase) (Fisher, 2017).

**TABLE 1:** Modified Racine’s scale.

Score	Behavioral changes
0	No changes in behavior
1	Myoclonic jerks with sudden and repetitive movement of the neck and head with or without tail stiffening
2	Unilateral or incomplete (Atypical) clonic seizure
3	Clonic seizure with forelimb clonus and rearing
4	Tonic-clonic seizure with an initial wild run and loss of righting reflex
5	Tonic-clonic seizure with full extension of fore and hind limbs

### 2.5 Sample preparation

After evaluation of seizure behavior, the blood samples were collected from the hearts of all rats under deep anesthesia using 80 mg/kg ketamine with 10 mg/kg xylazine (Abdulwahab et al., 2021; Azizi et al., 2018) into potassium EDTA-coated tubes and immediately centrifuged at 13,000 rpm for 15 min. Then, blood plasma was separated for biochemical analysis and stored in–80°C. In the following, the hippocampus and cerebellum of sacrificed rats were removed from all groups. One-half of the hippocampus and cerebellar hemisphere were washed with 0.9% saline, and kept at −80°C for mRNA expression study, while the other half were excised and fixed with 10% formalin (KalaZist, Iran) for histopathological observation (Kitazawa et al., 2005; Ren et al., 2020).

### 2.6 Histopathological analysis

Following post fixation, the rat brain samples underwent standard processing and were enclosed in paraffin. Thin sections measuring 5 µm were obtained at the hippocampus and cerebellum levels using a microtome from SLEE Medical CUT 6062, Germany. Sections were subsequently stained with H&E and examined under an Olympus Optical Co., LTD. light microscope (Tokyo, Japan) in × 40 and × 400 magnification for histological examination and count of dark neurons (Bancroft and Gamble, 2008). Dark neurons are damaged neurons that appear shrunken and dark under a microscope. They have condensed cytoplasm and pyknotic nuclei (Fujikawa et al., 2000). Scale bars were created manually using the scale bar tool in ImageJ software (version 1.5.3).

### 2.7 Measurement of GPx, SOD and MDA

The GPx activity kit (product No/SKU: KGPX96, Kiazist, Iran) relied on the reaction of reduced glutathione (GSH) with hydrogen peroxide (H_2_O_2_) producing H_2_O and oxidized glutathione (GSSG). Absorbance changes at 340 nm were recorded every 1 min for 5 min in Kinetic mode, and GPx activity was reported as nmol/min/mL of plasma.

The SOD activity kit (product No/SKU: KSOD96, Kiazist, Iran) determined the SOD’s ability to impede the conversion of resazurin to resorufin while reducing superoxide radicals generated by the xanthine/xanthine oxidase system. After the reaction, the absorbance of resorufin at 570 nm was measured, and for each sample the percentage of resorufin inhibition rate was calculated and converted to activity of SOD using the formula: (inhibition rate 50% = 1 U SOD activity).

The TBA assay kit (product No/SKU: KMDA96, Kiazist, Iran) was employed to gauge lipid peroxidation as per the manufacturer’s guidelines. This assay is grounded on the reactivity of MDA with TBA, forming a chromophore complex detectable at a wavelength of 532 nm. The concentration of MDA was determined based on a standard curve established using the MDA standard available in the kit and reported as nmol/mL of plasma.

### 2.8 Real-time quantitative PCR (RT-qPCR)

Analysis and amplification of RT-qPCR were carried out using the micPCR software version 2.12.6 (Bio Molecular Systems, Australia). The reaction included SYBR Green Master Mix, a specific primer pair (Sina Colon Co. Iran), which was designed with Oligo 7 software (Rychlik, 2007) based on the DNA sequences of GenBank (Table 2). RT-qPCR analysis was performed using a 20 µL reaction volume. The reaction blend comprises 10 µL of 2X SYBR Green PCR Master Mix, 1 µL of each forward and reverse primers (10 pmol), 1 µL of cDNA, and 7 µL of nuclease-free water (Sina Colon Co. Iran). Amplification settings were as follows: for *glyceraldehyde-3-phosphate dehydrogenase* (*Gapdh*) and *Nrf2*: 95°C for 5 min (holding), 40 cycles of 95°C for 15 s (denaturation), 58°C for 20 s (annealing), and 72°C for 20 s (extension). For *Hmox1*: 95°C for 5 min, 40 cycles of 95°C for 15 s, 55°C for 20 s, and 72°C for 20 s. The comparative cycle threshold (Ct) test was used to determine the relative expression of the examined mRNA gene. *Gapdh* was used as an endogenous housekeeping gene (reference gene) and the values were normalized to it.

**TABLE 2:** The primer sequence of the targeted gene.

Gene name	Primer sequence	GenBank accession number	Product length (base pair)
*Nrf2*	Forward: 5′- CACAAAAGACAAACATTCAAG -3′ Reverse: 5′- AAGTCATCAACAGGGAG -3′	NM-031789.2	120 bp
*Hmox1*	Forward: 5′- TTTTCACCTTCCCGAGCATC -3′ Reverse: 5′- ATCTCCAGAGTGTTCATGCG -3′	NM_012580	76 bp
*Gapdh*	Forward: 5′- AAGTTCAACGGCACAGTCAAG -3′ Reverse: 5′- CATACTCAGCACCAGCATCAC -3′	NM_017008	100 bp

*Gapdh* glyceraldehyde 3-phosphate dehydrogenase, *Nrf2* nuclear factor erythroid 2-related factor 2, *Hmox1* heme oxygenase 1.

### 2.9 Statistical analysis

We utilized the GraphPad Prism 9 statistical software (GraphPad Software, Inc., CA, United States) for all statistical analyses, presenting the outcomes as mean ± standard deviation (SD). For epileptic behavior analysis, histopathological quantification, oxidative stress biomarkers, and RT-qPCR data, the analysis involved one-way ANOVA with Tukey’s test as a *post hoc* assessment. Throughout our analysis, statistical significance was defined as P < 0.05.

## 3 Results

### 3.1 Effect of glycitin on PTZ induced seizure

**FIGURE 2:**
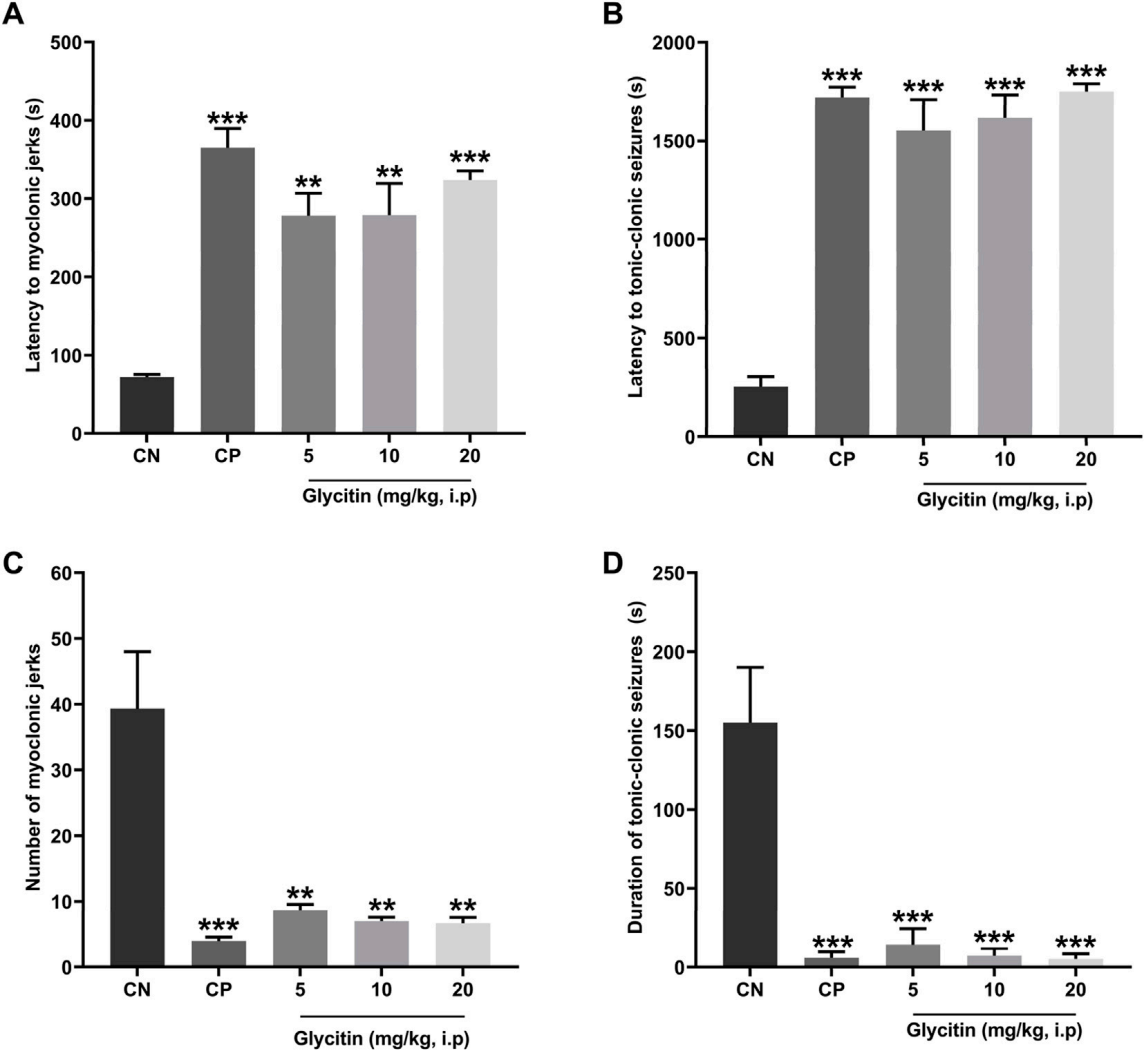
The effects of different doses of glycitin (5, 10, and 20 mg/kg) **(A)** latency to myoclonic jerks, **(B)** latency to tonic-clonic seizures, **(C)** number of myoclonic jerks, and **(D)** the duration of tonic-clonic seizures. Each column and hashtag denote statistically significant differences and bars represent the mean ± SD. n = 6. **P < 0.01 and ***P < 0.001 indicate a significant difference from the control negative (CN) group.

The seizure induced by a subthreshold dose of PTZ resulted in the prototyped movements such as myoclonic jerks and generalized tonic-clonic seizures, in all groups. The latency to the myoclonic jerks and tonic-clonic seizures in the CN group began after 72 ± 3.46 and 255 ± 50 s, respectively (Figures 2A, B). The number of myoclonic jerks and duration of the tonic-clonic seizures in the CN group were 39.33 ± 8.66 and 155 ± 35 s, respectively (Figures 2C, D). The latency to the myoclonic jerks and tonic-clonic seizures in the CP group began after 364.33 ± 25.30 and 1,560 ± 87.17 s respectively, which caused significant increase in comparison with the CN group (P < 0.001 and P < 0.001 respectively, Figures 2A, B). The number of myoclonic jerks and duration of the tonic-clonic seizures in the CP group was 4 ± 0.57 and 17.5 ± 2.5 s, respectively in comparison with the CN group (P < 0.001 and P < 0.001 respectively, Figures 2C, D). Glycitin administration significantly increased the onset to myoclonic jerks [F (4, 25) = 19.76, P < 0.001, Figure 2A] with statistical significance at 5 mg/kg (278 ± 28.59 s, P = 0.0014), 10 mg/kg (279 ± 40.15 s, P = 0.0013) and 20 mg/kg (323.33 ± 11.66 s, P < 0.001) compared with the CN group. Glycitin at the mentioned doses was able to increase the latency to the tonic-clonic seizures [F (4, 25) = 15.64, P < 0.001, Figure 2B] with statistical significance at 5 mg/kg (1,057.5 ± 247.5 s, P < 0.001), 10 mg/kg (1,250 ± 183.33 s, P < 0.001) and 20 mg/kg (1,650 ± 72.11 s, P < 0.001) compared with the CN group. The number of myoclonic jerks [F (4, 25) = 14.05, P < 0.001)] in the glycitin treatment groups was 8.66 ± 0.88, 7 ± 0.577, and 6.66 ± 0.88, respectively in comparison with CN group (P = 0.0019, P = 0.0012, and P = 0.0011 respectively, Figure 2C). Duration of the tonic-clonic seizures [F (4, 25) = 30.74, P < 0.001] in the glycitin treatment groups were 43 ± 17, 21.5 ± 2.5, and 15 ± 3 s, respectively in comparison with CN group (P < 0.001, P < 0.001, and P < 0.001 respectively, Figure 2D).

### 3.2 The effect of glycitin on the quantity of dark neurons in dentate gyrus (DG) region of hippocampus

**FIGURE 3:**
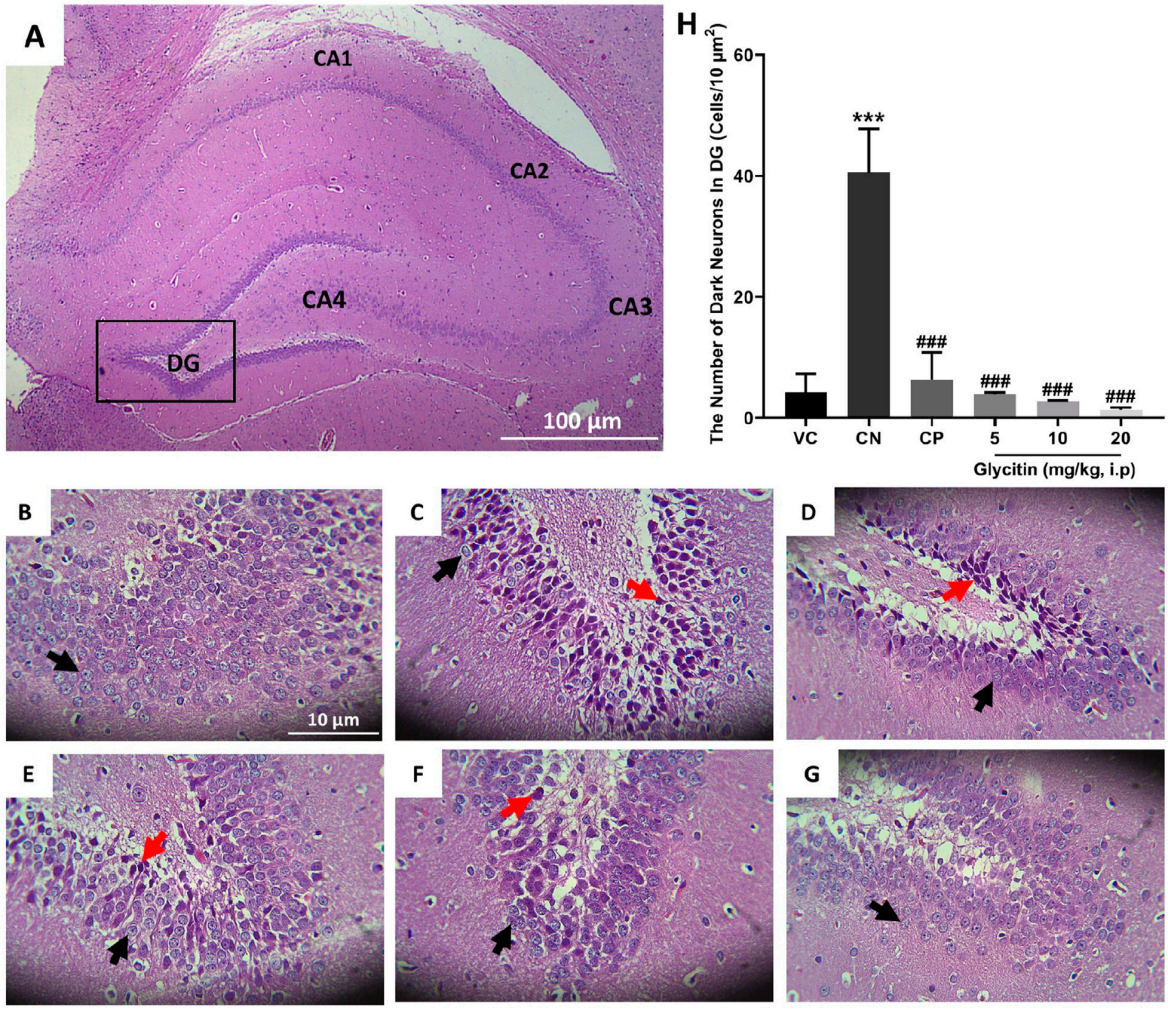
H&E staining of coronal hippocampal sections. **(A)** Section through VC group (×40) indicating the different regions of the hippocampal formation; The hippocampus itself consists of the Cornu Ammonis (CA) as CA1, CA2, CA3, and CA4 regions, and dentate gyrus (DG). **(B)** Section through the VC group (× 400), indicating normal granular cells (black arrow) in the DG region. **(C)** Section through the CN group, indicating dark neurons (red arrow) in granular cells in the DG region. Sections through CP group **(D)** and glycitin treated groups at the doses of 5 **(E)**, 10 **(F)**, and 20 **(G)** mg/kg showing prevention in the production of the dark neurons. **(H)** Comparing the dark neuron number in different groups. ***P < 0.001 in comparison with the VC group; ###P < 0.001 in comparison with the CN group.

The number of dark neurons present in the DG region of the rat hippocampus was counted for each study group. The mean number of dark neurons significantly increased in the CN group (40.55 ± 7.22/10 μm^2^) in comparison with the VC group (P < 0.001, Figures 3A–C, H) and in the CP group (6.33 ± 4.50/10 μm^2^) in comparison with the CN group (P < 0.001, Figures 3C, D, H). A significant reduction was counted in the mean number of dark neurons in the groups receiving glycitin [F (5, 30) = 16.76, P < 0.001, Figures 3C, E, F, H] with statistical significance at 5 mg/kg (3.88 ± 0.29/10 μm^2^, P < 0.001) and 10 mg/kg (2.66 ± 0.15/10 μm^2^, P < 0.001) compared with the CN group. 20 mg/kg of glycitin illustrated the most significant decrease on the number of dark neurons (1.33 ± 0.38/10 μm^2^) in comparison with the CN group (P < 0.001, Figures 3C, G, H).

### 3.3 The effect of glycitin on the quantity of dark neurons in Purkinje cell layer of cerebellar cortex

**FIGURE 4:**
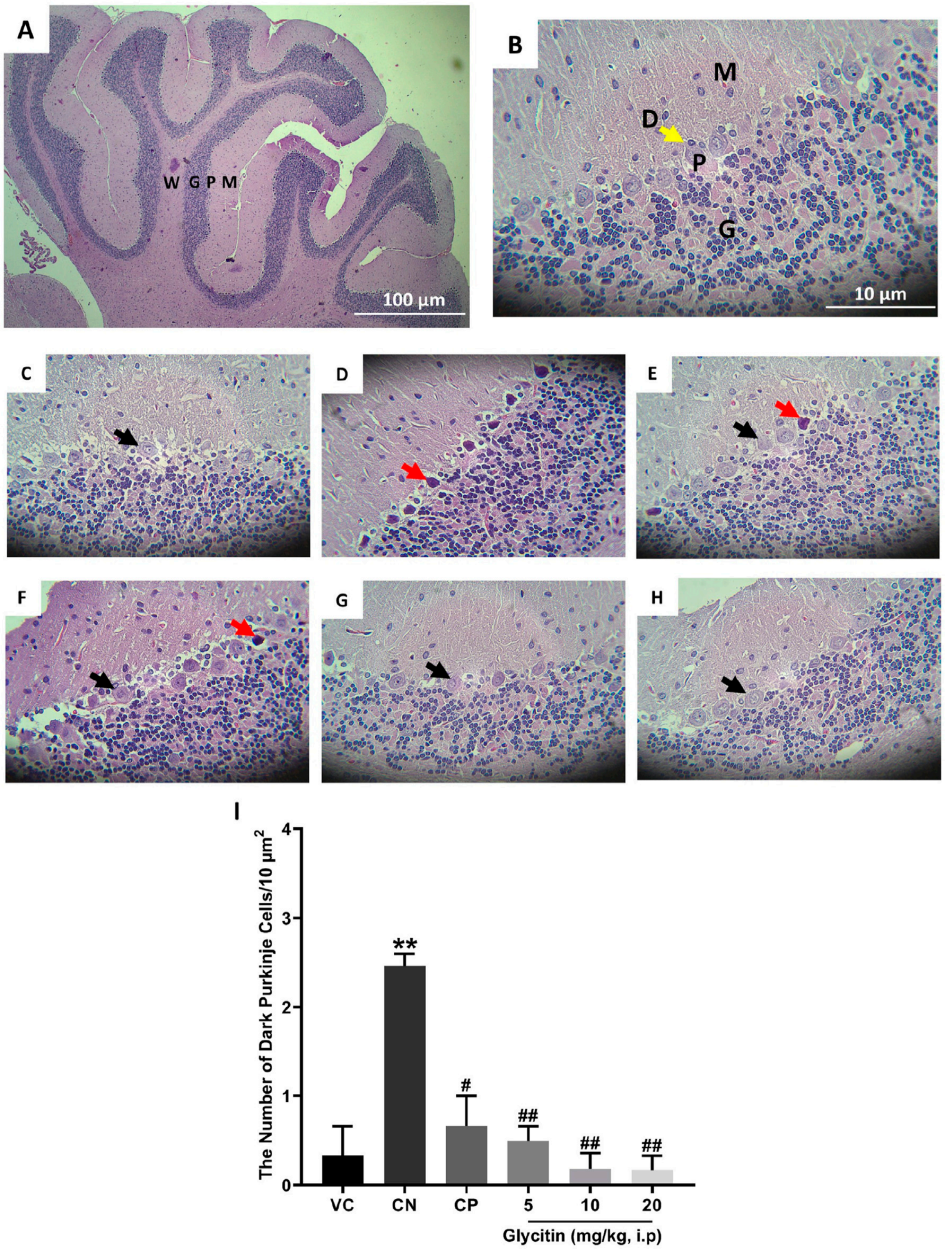
H&E staining of the rat cerebellar cortex in the different study groups. **(A)** Section through VC group (× 40) showing the granular cell layer (G), Purkinje cell layer (P), molecular cell layer (M), and the medulla which is formed of white matter fibers (W). **(B)** Section through the VC group (× 400), showing granular layer (G), Purkinje cell (P), molecular layer (M), and dendrites (D, yellow arrow). **(C)** Section through the VC group (× 400), showing normal Purkinje cells (black arrow). **(D)** Section through the CN group, showing dark neurons (red arrow) in Purkinje cell layer. Sections through CP group **(E)** and glycitin treated groups at the doses of 5 **(F)**, 10 **(G)**, and 20 **(H)** mg/kg showing prevention in the dark neuron’s formation to some extent. **(I)** Comparing the dark neuron number in different groups. **P < 0.01 in comparison with the VC group; #P < 0.05 and ##P < 0.01 in comparison with the CN group.

The H&E stained sections in the rat’s cerebellum of the VC group showed the characteristic structure of the cerebellar cortex, consisting of three layers: the granular cell layer (G), Purkinje cell layer (P), and molecular cell layer (M). The medulla, which consists of white matter fibers (W), was also seen (Figures 4A, B). In the Purkinje cell layer, the mean number of dark neurons significantly increased in the CN group (2.465 ± 0.135/10 μm^2^) in comparison with the VC group (P = 0.0049, Figures 4C, D, I). Compared with the CN group, the number of dark neurons in the Purkinje cell layer was notably reduced in the CP group (0.665 ± 0.335/10 μm^2^ with P = 0.0117) and glycitin treatment groups [F (5, 30) = 14.16, P = 0.0029] at doses of 5, 10, and 20 mg/kg (0.495 ± 0.165/10 μm^2^ with P = 0.0075, 0.18 ± 0.18/10 μm^2^ with P = 0.0035, and 0.165 ± 0.165/10 μm^2^ with P = 0.0033 respectively, Figures 4E–I).

### 3.4 Effect of glycitin on oxidative stress markers

**FIGURE 5:**
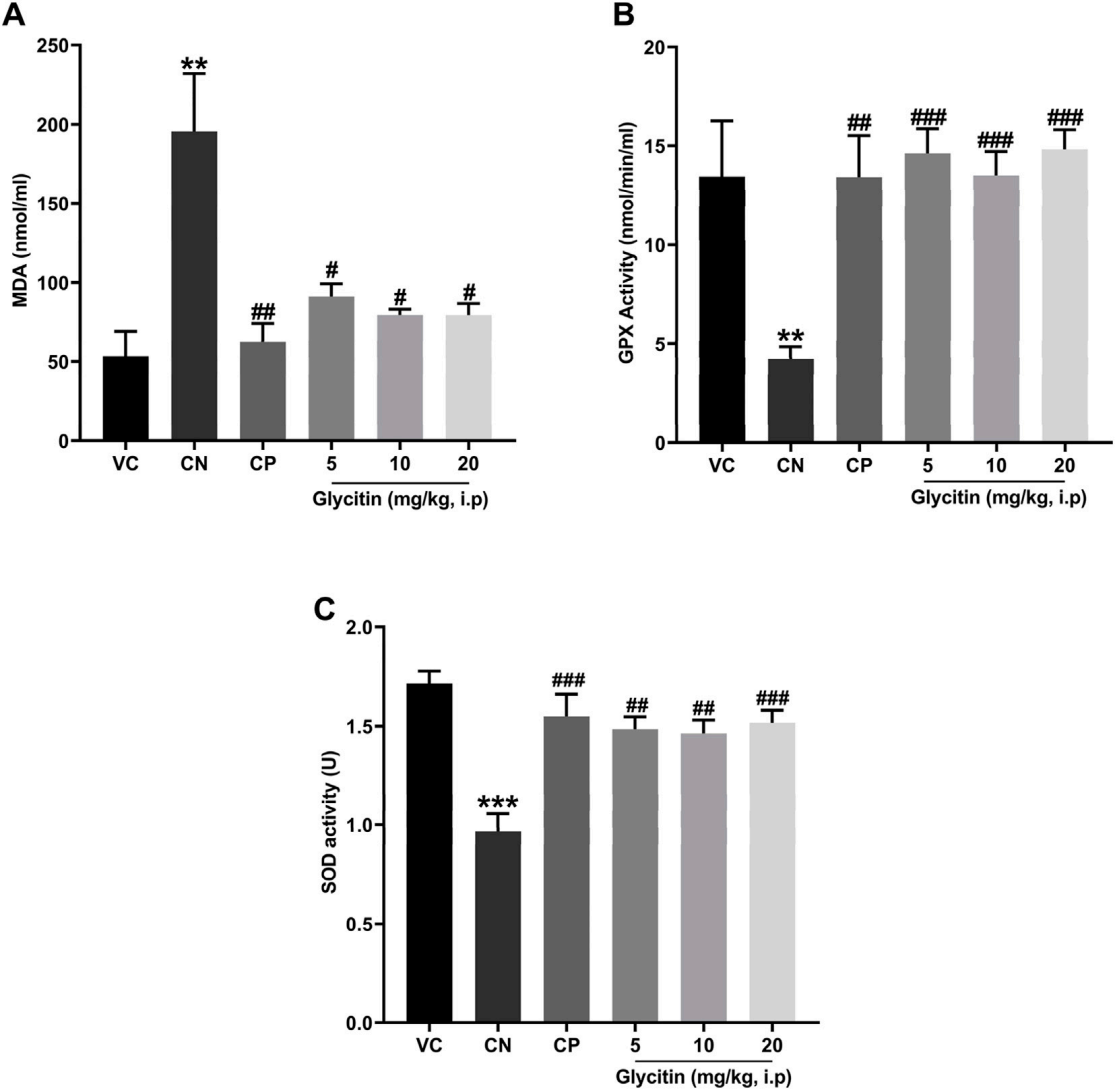
Comparison of the **(A)** the MDA levels and **(B)** GPx activity and **(C)** SOD activity of plasma between the studied groups. Data are expressed as mean ± SD. n = 6. **P < 0.01 and ***P < 0.001 indicate a significant difference from the VC group. #P < 0.05, ##P < 0.01, and ###P < 0.001 indicate a significant difference from the CN group.

The analysis of oxidative stress markers demonstrated that the concentrations of MDA were significantly higher in the CN group in comparison with the VC group (P = 0.0043, Figure 5A). Pretreatment with PB (30 mg/kg) and glycitin [F (5, 30) = 8.997, P = 0.0039] at the doses of 5, 10 and 20 mg/kg (P = 0.0026, P = 0.0262, P = 0.0146, and P = 0.0145 respectively, Figure 5A) remarkably reduced the MDA level in comparison with the CN group. There was statistically decreased activity of GPx and SOD observed in the CN group in comparison with the VC group (P = 0.0059 and P < 0.001 respectively, Figures 5B, C). GPx showed significantly elevated activity in both CP (P = 0.0026, Figure 5B) and glycitin treatment groups (F (5, 30) = 8.316, P < 0.001) at the doses of 5, 10 and 20 mg/kg (P < 0.001, P < 0.001, and P < 0.001 respectively, Figure 5B) in comparison with CN group. The SOD enzyme activity was also significantly higher in both CP (P < 0.001) and glycitin treatment groups [F (5, 30) = 9.130, P < 0.001] at the doses of 5 and 10 mg/kg (P = 0.0012 and P = 0.0019 respectively, Figure 5C) in comparison with CN group. The dose 20 mg/kg of glycitin revealed the most significant increase in the activity of SOD enzyme compared with the CN group (P < 0.001, Figure 5C).

### 3.5 Upregulatory effect of glycitin on the expression of *Nrf2* and *Hmox1* genes

**FIGURE 6:**
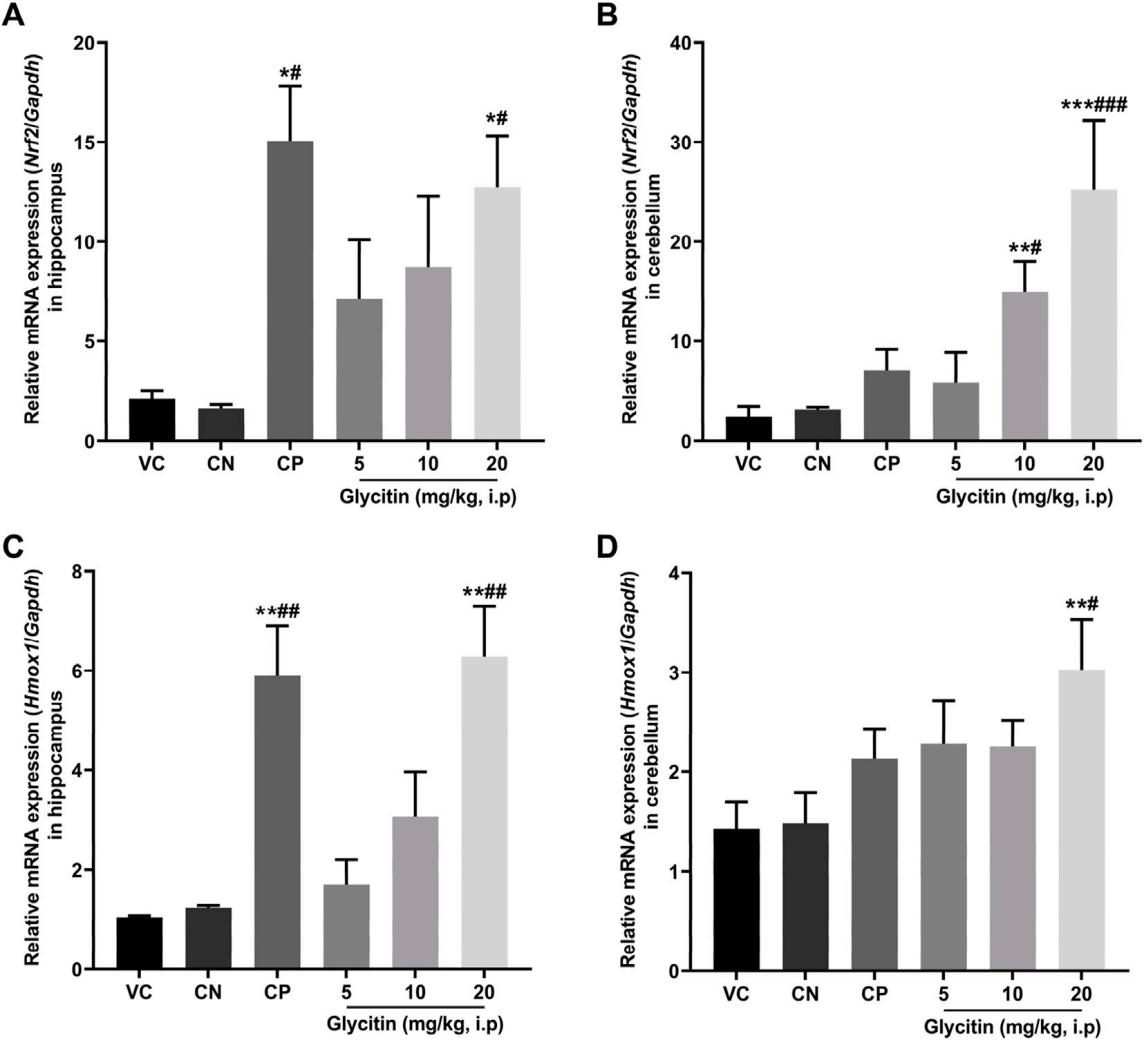
Effect of glycitin on the mRNA expression of *Nrf2* and *Hmox1* genes on the rat’s hippocampus and cerebellum. The mRNA expression of target genes evaluated by RT-qPCR. **(A)** *Nrf2* mRNA expression in hippocampus **(B)** *Nrf2* mRNA expression in cerebellum **(C)** *Hmox1* mRNA expression in hippocampus **(D)** *Hmox1* mRNA expression in cerebellum. Data are expressed as mean ± SD. n = 6. *P < 0.05, **P < 0.01 and ***P < 0.001 indicate a significant difference from the VC group. #P < 0.05, ##P < 0.01, and ###P < 0.001 indicate a significant difference from the CN group.

In response to oxidative stress, the mRNA expression of *Nrf2* and its downstream gene *Hmox1* in the rat hippocampus and cerebellum, were measured by Real-time PCR. The results illustrated that the mRNA expression of *Nrf2* was notably increased in the CP group (P = 0.0104) and 20 mg/kg glycitin treatment group (P = 0.0302, Figure 6A) in the hippocampus compared with the CN group [F (5, 30) = 7.936, P = 0.0058]. Glycitin at the doses of 10 and 20 mg/kg [F (5, 30) = 20.25, P < 0.001], especially at the concentration of 20 mg/kg, illustrated a significant increase in the mRNA expression of *Nrf2* in the cerebellum compared with the CN group (P = 0.0118 and P < 0.001 respectively, Figure 6B). In addition, results revealed that the mRNA expression of *Hmox1* was notably increased in the CP group (P = 0.0024) and 20 mg/kg glycitin treatment group (P = 0.0026, Figure 6C) in the hippocampus compared with the CN group [F (5, 30) = 21.01, P < 0.001]. Exposure to 20 mg/kg of glycitin notably increased [F (5, 30) = 7.084, P = 0.0082] the mRNA expression of *Hmox1* in the cerebellum compared with the CN group (P = 0.0102, Figure 6D).

## 4 Discussion

Epilepsy is a neurological disorder that has a significant impact worldwide (Scharfman, 2007). Studies have indicated that traditional isoflavones from soy are capable of reducing seizures by inhibiting oxidative stress (Elsayed et al., 2018; Hu et al., 2021; Kazmi et al., 2020). Based on this knowledge, we propose that glycitin, another soy isoflavone (Chen et al., 2019) not extensively studied for its anticonvulsant effects, may also have the potential for alleviating convulsions induced by PTZ through oxidative stress suppression. To test this hypothesis, we used a PTZ kindling model to induce chronic epilepsy in rats. PTZ kindling is a widely accepted method for studying epileptogenesis in experimental animals (Gao et al., 2018; Liu et al., 2018a). Our observations revealed that in the groups treated with glycitin the onset of the myoclonic jerks and tonic-clonic seizure was associated with a greater delay than the CN group. In addition, a reduction in the number of myoclonic jerks and the duration of tonic-clonic seizure were observed in the glycitin treatment groups than the CN group. These results strongly indicate the anticonvulsant properties of glycitin.

Persistent epilepsy and seizures have the potential to result in neuronal damage (Lucke-Wold et al., 2015). Neuronal loss can exacerbate the seizure severity (Zhang et al., 2002), and crucially, neuronal damage is directly associated with the initiation of epilepsy (Sayin et al., 2003). The hippocampus has long been a pivotal focus in epilepsy research and observations have shown a reduction in the size of hippocampi in epileptic patients’ brains (Pitkänen and Sutula, 2002). One of the key mechanisms underlying the dark and shrunken morphology of granule cells in the DG during epileptic episodes is excitotoxicity, primarily mediated by excessive glutamate release (Wang and Qin, 2010). During seizures, elevated levels of glutamate can lead to overstimulation of glutamate receptors, particularly N-methyl-D-aspartate (NMDA) receptors (Barker-Haliski and White, 2015; Vasilev et al., 2018). This overstimulation results in increased intracellular calcium levels, leading to neuronal injury and apoptosis (Barker-Haliski and White, 2015; Khan et al., 2021). The DG serves as a critical gateway to the hippocampus, acting as a crucial controller for cortical input to the limbic system (Dengler and Coulter, 2016). Its low excitability not only limits the transmission of abnormal cortical activity into the hippocampus but also governs seizure activity in conditions like epilepsy, known as “dentate gating” (Dengler and Coulter, 2016). When this regulatory function is impaired during the development of epilepsy, the potent excitatory impact of DG on downstream targets within the hippocampus may enable the propagation and intensification of synchronous pathological activity throughout the limbic system, potentially triggering increased seizure propensity (Dengler and Coulter, 2016). In addition to the hippocampal DG region, there is evidence to suggest the cerebellum is affected during seizures, and cerebellum damage is observed in epilepsy patients (Streng and Krook-Magnuson, 2021). *Postmortem* examination of patients with chronic epilepsy reveals a noticeable atrophy of cerebellar Purkinje cells (Crooks et al., 2000; Ibdali et al., 2021). Therefore, after evaluating the anticonvulsant properties of glycitin, we conducted further investigations on its impact on hippocampal DG region and cerebellar Purkinje neuronal damage in PTZ kindled rats using H&E staining. A majority of the neurons in the hippocampal DG region in the CN group exhibited damage. The resulting cellular damage manifested as a darkened and shrunken appearance due to chromatin condensation and cytoplasmic alterations, indicative of necrosis or apoptosis (Covolan et al., 2000; Yardımoglu et al., 2007). However, rats treated with glycitin exhibited a reduction in their hippocampal dark neurons. Similar to the hippocampus, a significant portion of cerebellar Purkinje cells in the CN group exhibited dark appearance with a significant cell shrinkage and cytoplasmatic condensation, which is indicative of cellular stress or degeneration. In contrast, rats treated with glycitin showed a decrease in their cerebellar Purkinje dark neurons. These findings indicate that glycitin has the potential to prevent hippocampal DG region and cerebellar Purkinje neuronal damage induced by PTZ kindling. Data from the present study clearly indicate that glycitin can guard against PTZ induced seizures and prevent damage to the hippocampal DG region and cerebellar neurons.

In a recent study, it was highlighted that oxidative stress induced by epilepsy has the potential to initiate a chain reaction, leading to extensive neuronal damage in the brain, thereby exacerbating epileptic seizures (Grewal et al., 2017). Oxidative stress has the potential to intensify neuronal hyperactivity and excitotoxicity, particularly through the glutamatergic dysfunction and overactivation of glutamate receptors, consequently heightening susceptibility to epilepsy (Borowicz-Reutt and Czuczwar, 2020; Chen et al., 2023; Puttachary et al., 2015). Our investigation unequivocally illustrated the anti-seizure and neuroprotective effects of glycitin. This is attributed to glycinin’s intrinsic nature as a natural antioxidant (Chen et al., 2019). Lee et al. (2005), demonstrated that glycitin exhibits strong antioxidant properties, as determined by the ferric reducing-antioxidant power (FRAP) assay and Zang et al. (2015), suggested that the regulation of oxidative stress by glycitin was related to the suppression of adipose tissue and the progression of diabetes. Consequently, we delved into exploring the impact of glycitin on oxidative stress caused by PTZ kindling to better comprehend the neuroprotective potential of glycitin. Our findings revealed that PTZ kindling led to an increase in the MDA content while reducing the GPx and SOD activities in the rat plasma. Notably, glycitin exhibited the capability to counteract the effects of PTZ. These outcomes emphasize the antioxidative potential of glycitin in the PTZ induced epilepsy rat model, suggesting that this effect may have a pivotal role in the neuroprotective actions of glycitin. Furthermore, the modulation of oxidative stress levels can influence the homeostasis of glutamate in the synaptic cleft, potentially preventing the pathological hyperexcitability associated with epilepsy (Al-Shorbagy et al., 2021; Yang et al., 2020). This protective effect may be particularly relevant in conditions where glutamate signaling is disrupted due to oxidative damage (Roganovic et al., 2019).

Increased GPx and SOD activities in the glycitin treated rats may have resulted due to increased expression of *Nrf2* and *Hmox1* (Singh et al., 2018). Nrf2/HO-1 antioxidant signaling pathway protects neurons from oxidative damage associated with excessive glutamate signaling (Khan et al., 2021). By mitigating oxidative stress, the Nrf2/HO-1 pathway helps maintain neuronal health and prevents excitotoxicity, thereby supporting proper glutamatergic neurotransmission and reducing the risk of neurodegenerative processes (Khan et al., 2021; Parfenova et al., 2006). The present study results indicated that 20 mg/kg of glycitin notably upregulated the levels of *Nrf2* and *Hmox1* expression in the hippocampus and cerebellum of the rat after PTZ kindling. Our results suggested that glycitin exerts a protective effect on PTZ induced epilepsy by obvious upregulation of *Nrf2* and *Hmox1* genes expression.

Overall results of this study, in addition to being consistent with the results of previous studies, provide significant evidence that glycitin can ameliorate PTZ induced seizures by regulation of oxidative stress, *Nrf2*/*Hmox1* expression, and neuronal damage. It is important to note that while phenobarbital has been widely used for decades in the treatment of epilepsy, it is associated with significant side effects such as sedation, cognitive impairment, and risk of dependence (Kubova, 2016). In contrast, glycitin, as an herbal compound, has shown promising results in our study for its anticonvulsant properties with similar or even better effects in controlling epilepsy compared to phenobarbital. Given the potential long-term side effects associated with anti-seizure medications, and the absence of acute complications observed during our experiment, glycitin appears to be a promising candidate for experiments and laboratory tests.

## 5 Conclusion

The current study demonstrates that glycitin has inhibitory effects on PTZ induced epilepsy in rats, reducing associated symptoms. It shows that glycitin exerts its anti-epileptic effects by modulating oxidative stress, Nrf2/HO-1 signaling, and neuronal damage in brain tissue. However, further research is needed to fully understand the molecular mechanisms underlying its actions.

## Data Availability

The original contributions presented in the study are included in the article/supplementary material, further inquiries can be directed to the corresponding author.
